# PReS-FINAL-2003: Remission and predictors of persistent disease activity at 30 years follow-up in a Norwegian cohort of juvenile idiopathic arthritis patients

**DOI:** 10.1186/1546-0096-11-S2-O6

**Published:** 2013-12-05

**Authors:** AM Selvaag, HA Aulie, V Lilleby, B Flatø

**Affiliations:** 1 Department of Rheumatology, Oslo University Hospital, Rikshospitalet, Oslo, Norway

## Introduction

Long-term studies of remission rates in juvenile idiopathic arthritis (JIA) are few and difficult to compare because of different definitions of remission.

## Objectives

To assess disease activity in a previously studied cohort of JIA patients after 30 years of disease duration and reveal predictors of persistently active disease.

## Methods

A total of 259 patients with JIA, first referred to our hospital from 1980 to1985, were reexamined clinically after median 15 years of disease duration, and by mailed questionnaires after median 23 years. These patients were invited to attend the present study. All patients were assessed by questionnaires, and those with signs of active disease after 15, 23 and/or 30 years were invited to a clinical reexamination. Inactive disease and remission were defined according to the recently developed preliminary criteria for clinical remission in JIA. Logistic regression analyses were used to assess predictors of active disease.

## Results

One hundred and seventy-one patients (67%) were included in the study. They were examined after a median of 30 (range 21-40) years of disease duration, median age was 39 (range 28-45) years and 74% were females. After 30 years, 59% of the patients were in clinical remission off medication, 7% were in remission on medication and 34% had persistently active disease compared with 57%, 10%, and 33%, respectively, after 15 years.

**Table 1:** 

Disease activity at 15 years follow-up		Not in remission at 30 years	Remission on med. at 30 years	Remission off med. at 30 years
Not in remission	57 (33%)	37	9	11
Remission on med.	17 (10%)	9	2	6
Remission off med.	97 (57%)	12	1	84
Total	171 (100%)	58 (34%)	12 (7%)	101 (59%)

med.: medication

Thirty-seven of 57 patients (65%) with active disease at 15 years follow-up had active disease at 30 years follow-up, and 20 patients (35%) went into remission on or off medication. Eighty-four of 97 patients (87%) in remission off medication remained in this category from 15 to 30 years follow-up. Patients in remission on medication at 15 years (n = 17) tended to flare or go into remission off medication. In total 121/171 patients (71%) had an unchanged category of disease activity from 15 to 30 years follow-up. Predictors of persistently active disease at 30 years follow-up were: being diagnosed with a JIA subgroup other than persistent oligoarticular and systemic JIA (OR 4.1, 95%CI 1.5-11.5, p = 0.006), being DR1 positive (OR 8.3, 95%CI 2.3-30.3, p = 0.001), a short duration of total time in remission (OR 9.0, 95% CI 3.0-26.7, p < 0.001), and not being in remission at 15 years follow-up (OR 13.7, 95%CI 4.9-38.4, p < 0.001), Nagelkerkes R^2 ^= 65%.

## Conclusion

The overall remission rates were stable between 15 and 30 years, even though one third of the patients changed category of disease activity. JIA subgroup, genetic factors and time without remission were important predictors of long-term outcome.

## Disclosure of interest

None declared.

